# Activity ordering task: conceptualization and development of a novel context-based working memory task with a metacognitive facet

**DOI:** 10.1590/2317-1782/20242024041en

**Published:** 2024-10-11

**Authors:** Nidhi Lalu Jacob, Gagan Bajaj, Aysha Rooha, Vinitha Mary George, Jayashree S. Bhat

**Affiliations:** 1 Department of Audiology and Speech Language Pathology, Kasturba Medical College Mangalore, Manipal Academy of Higher Education – Manipal (Karnataka), India.; 2 Department of Audiology and Speech Language Pathology, National Institute of Speech & Hearing – Trivandrum (Kerala), India.; 3 Department of Audiology and Speech Language Pathology, Nitte Institute of Speech and Hearing, Nitte Deemed to be University Deralakatte – Mangalore (Karnataka), India.

**Keywords:** Everyday Working Memory, Activity Ordering Task, Metacognition, Indian Adults, Context-based Task

## Abstract

**Purpose:**

The aim of the present study was to conceptualize and develop a novel context-based working memory (WM) task which would possess the potential to assess the context-based WM in an Indian scenario and incorporate a task-linked metacognitive facet to understand an individual's self-perception of performance.

**Methods:**

Employing the ADDIE instructional design model, comprising Analysis, Design, Development, Implementation and Evaluation phases, the present study focused on the initial four phases. In the Analysis-phase, the needs and objectives for creating a context-based WM task were identified. Design-phase included task conceptualization and content validation of the conceptualized context-based WM task. In the Develop-phase, the designed context-based WM task was developed in an animated video format. Five content experts and fifteen lay experts were involved in the content validation process. In the Implementation-phase, a pilot study was done on ten adults to assess the feasibility of the novel context-based WM task.

**Results:**

The task obtained good content validation index and understandability scores on the Patient Education Materials Assessment Tool. Preliminary data trends from the implementation-phase revealed the task's potential to detect age-related WM differences. Significantly correlated with established WM tasks, the novel Activity Ordering Task (AOT) effectively measured WM-spans. Observed discrepancies between performance and prediction/postdiction spans during metacognitive facet administration highlighted the AOT's utility in evaluating metacognition.

**Conclusion:**

Addressing the limitations of context in traditional tasks, the AOT appears to be a promising tool that not only measures WM but also assesses metacognition, thereby enhancing its utility beyond an everyday WM assessment.

## INTRODUCTION

In navigating the intricacies of everyday life, working memory (WM) serves as a cognitive cornerstone, orchestrating the conscious retention and manipulation of information. WM facilitates cognitive-communicative activities such as recalling shopping lists, executing step-by-step cooking recipes, comprehending directions in unfamiliar routes, engaging in phone conversations, multitasking seamlessly, paying bills, planning events, remembering appointments, solving problems effectively, and managing information^(1)^. WM is widely recognized as the domain of human cognition involved in the temporary storage and manipulation of information^(2)^. It plays a pivotal role in mediating the processing of stored or incoming information, bridging its utilization for diverse cognitive goals such as orientation, reasoning, language processing, planning, and spatial processing^(3)^. Performance on WM tasks reflect the interplay between WM capacity and executive function components that govern attention, switching and interfacing with long-term memory^(4)^. Extensive research indicates that WM declines with age, emphasizing the importance of evaluating it for the early identification and intervention of cognitive disorders.

Over the years, laboratory-based tasks have been the basis of assessing WM capacity and performance. These assessments encompass a diverse array of tasks aimed at evaluating critical aspects of WM maintenance, manipulation and updating capabilities. Complex Span tasks such as Reading Span, Listening Span, Operation Span, Rotation Span, Digit Span and Sentence Ordering tasks^(5-7)^ assess information processing and manipulation. n-back tasks^(8)^ measure the ability to compare and recall stimuli across different time intervals, while memory updating tasks^(7)^ focus on assessing the manipulation and updating of information within WM. Sorting Span tasks^(8)^ aim to gauge the efficiency in organizing and managing information.

### Laboratory-based assessment tasks

As we delve into the complexities of human cognition, a growing body of research has illuminated the limitations inherent in relying solely on laboratory-based tasks to understand WM’s nuances. Traditional laboratory-based cognitive tasks often inadequately replicate the multifaceted demands of real-life WM, leading to a disparity between laboratory performance and actual functional capacity^(9)^. Studies have highlighted this discrepancy, emphasizing the 'lab-life gap' and the constraints of such assessments^(10)^. Moreover, the rigidity of laboratory-based tasks and their limited variability starkly contrast with the dynamic nature of real-world contexts, thus questioning their ecological validity^(11)^. Despite acknowledging the value of traditional laboratory-based tasks, it is vital to recognize their limitations in capturing the intricate interplay between cognitive abilities and the complexities of real-life scenarios^(12)^.

In the realm of cognitive evaluation, assessments that mimic the challenges encountered in daily life are considered superior predictors of an individual's functional performance. Studies have demonstrated that cognitive abilities appear unaffected by age when evaluated within realistic memory contexts, while traditional paper-pencil or computer-based tasks tend to disadvantage older adults (OAs), underlining the importance of reality-based assessments that mirror real-life experiences^(13)^. Compared to conventional laboratory-based tasks, context-based tasks embedded within real-life situations tend to elicit heightened test motivation and greater participant involvement, likely stemming from a more favorable emotional reaction and inherent drive to complete the tasks^(10)^. Given the shortfall of laboratory-based tasks in measuring cognitive constructs relevant to everyday life, there is a growing demand for ecologically valid measures that capture real-world performance^(13)^.

### Shift towards ecologically valid measures

Recognizing the limitations of traditional laboratory-based cognitive tasks, several authors have developed context-based ecologically valid tasks to bridge the gap. These ecologically valid tasks have shown efficacy in predicting real-world performance and identifying cognitive impairments that may be overlooked by conventional laboratory-based tasks^(11)^. Notable examples include the 'Overnight Trip Task'^(9)^, 'Shopping Mall Task'^(14)^ and 'Breakfast Task,'^(15)^ which have been developed to assess cognitive abilities crucial for real-world functioning, emphasizing the introduction of realism. However, despite these developments, there remains a shortage of context-based assessments tailored to specifically evaluate WM. Moreover, cultural backgrounds play a crucial role in how individuals interpret task performance, shape neural activity and approach cognitive tasks. The cultural background of individuals determines the relevance and significance of different situations, providing the foundation for cognitive testing procedures^(16)^. The presence of clear cultural elements in cognitive tests emphasizes their sensitivity, creating challenges for the psychometric properties, especially when dealing with diverse populations, underscoring the necessity of considering cultural variations when applying standardized norms^(17)^.

In the specific context of developing a context-based WM assessment task tailored for Indian adults, there is a critical gap in cognitive assessments for adults due to the multifaceted nature of demographic diversity, encompassing language, religion and cultural factors. This myriad of factors necessitates a nuanced approach, considering within-group differences^(18)^. Current assessments fall short in providing the necessary specificity for seemingly homogenous populations, accentuating the pressing need to craft context-based tasks tailored explicitly for the Indian population.

### Role of metacognition in everyday lives of adults

Besides the imperative of creating context-based cognitive tasks, incorporating a metacognitive component within the task stands as a crucial attribute that can significantly augment their overall utility. Metacognition, described as 'thinking about thinking,' is essential for adult life, fostering skills in learning, critical thinking and decision-making^(19)^. It involves awareness of one's capabilities, strategy selection and monitoring of progress, contributing to successful task accomplishment^(20)^. Metacognition is crucial for adaptive decision-making, influences learning strategies, enhances cognitive efficiency and mitigates biases^(19)^. As individuals age, metacognition becomes pivotal in adapting to cognitive changes and maintaining an optimal quality of life, impacting functional independence and autonomy^(21)^.

As researchers increasingly recognize the role of metacognition in effective cognitive functioning, the integration of metacognitive components in modern laboratory cognitive assessments is gaining prominence. Several WM tasks have incorporated a metacognitive component, including listening span task, reading span task, digit ordering task, n-back task, visuospatial WM tasks and the operation span task^(5,7,8)^.

In literature, metacognition is generally investigated using both offline and online measures. Offline measures delve into the broader landscape of metacognitive awareness, employing tools such as questionnaires and self-report measures. These retrospective evaluations provide a panoramic view, capturing metacognitive insights independent of specific cognitive tasks^(22)^. Online measures of metacognition provide a dynamic perspective across cognitive tasks, incorporating prospective, concurrent and retrospective assessments. Prospective measures, administered before a cognitive task, encompass evaluations like feelings of confidence^(23)^ and performance estimation discrepancy based on prediction ratings^(24)^. Concurrent measures involve real-time evaluation of metacognition during task execution, often utilizing methods like think-aloud protocols^(25)^. Retrospective measures delve into post-task metacognitive assessment, employing approaches such as retrospective confidence judgment and performance estimation discrepancy measures based on postdiction ratings^(24)^.

The performance discrepancy method which has been extensively utilized^(24,26)^, assesses an individual's awareness or perceptions of specific abilities closely related to a given task. It operates as a prospective measure when computed based on predictions and transforms into a retrospective measure when derived from postdictions. In addressing a crucial gap in research, it is essential to highlight the scarcity of context-based tasks incorporating metacognitive components, particularly those specifically designed for the Indian population.

### Ecologically valid WM tasks with a metacognitive facet: a perspective on human communication and its disorders

WM plays a crucial role in human communication, facilitating the processing and understanding of information in real-time, retaining and manipulating details, language comprehension, aiding in language production and enhancing social interactions by interpreting cues and formulating appropriate responses^(1-3),^ Deficits in WM can lead to impairments in various linguistic functions, including the use of figurative language, context integration in conversations, narrative coherence, and verbal fluency, all of which are common in neurogenic communication disorders. Studies involving adults with dementia, aphasia, and traumatic brain injury have highlighted the significance of WM in mediating language comprehension, expression, and overall communication^(27)^. Metacognition associated with WM also plays a crucial role in driving everyday communication as it can drive individuals to either overestimate or underestimate their abilities, potentially resulting in fatal errors or underperformance in diverse communicative situations^(20)^. Therefore, ecologically valid tasks aimed at assessing WM and the associated metacognitive processes in individuals with typical and disordered communication could prove vital for gaining a comprehensive understanding and effectively managing these conditions.

### Present research

The evident need for a culturally grounded context-based task intertwining WM and metacognition among adults highlights the urgency in developing a novel assessment task relevant in Indian context. Such a task would open avenues for groundbreaking insights into the intersection between WM and metacognition in daily life, contributing significantly to the advancement of cognitive research. Consequently, the present study aimed at conceptualizing and developing a novel context-based WM task which would possess the potential to assess the context-based WM in an Indian scenario and incorporate a task-linked metacognitive facet to understand an individual's self-perception of performance. The specific objectives of the present study were firstly, to systematically devise and validate the content of a novel context-based WM task tailored for Indian adults and secondly, to examine its feasibility on a small sample of neurotypical Indian adults.

## METHOD

The present study is a methodological study aimed at conceptualizing, developing and validating a novel context-based WM task with a metacognitive facet. The study followed the ADDIE model of instructional design, an acronym representing its five constituent steps: Analysis, Design, Development, Implementation and Evaluation phases^(28)^. The present study focused on the first four phases which involve identifying the need, designing, development and testing the feasibility of the context-based WM task. The Evaluation phase planned to be conducted as a separate study would assess the psychometric properties of the newly developed context-based task for assessing WM. Ethical approval was obtained from the Institutional Ethics Committee (IECKMCMLR-08/2021/263, IECKMCMLR-05/2023/269).

### Participants

The study included five content experts and 15 neurotypical lay experts in the design and develop phase of the novel context-based WM task. The content experts were Speech-Language Pathologists (SLPs) with over five years of expertise in cognitive science. The lay experts consisted of five young adults (YAs) (18-40 years), five middle-aged adults (MAAs) (41-65 years), and five OAs (>65 years) who had no background in health or allied health fields. The involvement of lay experts ensured a precise representation of the target population, contributing valuable input and feedback that enhanced the task's relevance and usability across different age groups. To assess the feasibility of the novel context-based WM task (Implementation phase), ten neurotypical adults (five YAs, five MAAs) who were not part of the design and develop phase of the task were included. Participants eligible for inclusion in the design, development and implementation phases were those who scored above 26 on the Mini-Mental State Examination^(29)^ and had no history of neurological or psychological disorders. Further, the socioeconomic status of all participants was determined as middle class using the Modified Kuppuswamy scale^(30)^. Participant’s English proficiency was verified by ensuring a minimum proficiency score of 'Seven' on the Language Experience and Proficiency Questionnaire (LEAP-Q)^(31)^. All participants signed and provided informed consent. Detailed demographic information for all participant groups can be found in table 1.

**Table 1:** Participant characteristics

Attribute	Participants for Content Validation (Design and Develop Phase)				Participants for pilot study (Implementation Phase)	
	Content Experts (N=5)	Lay experts (N=15)			YA (N=5)	MAA (N=5)
	SLP	YA (n=5)	MAA (n=5)	OA (n=5)		
Mean Age	30±4.24 years	19.6+2.07 years	54.4+5.36 years	71.4+5.68years	22+1.58 years	51.8+7.79 years
Gender	Male: n=1	Male: n=1	Male: n= 3	Male: n=2	Male: n=0	Male: n=2
	Female: n=4	Female: n=4	Female: n=2	Female: n=3	Female: n=5	Female: n=3
Mean LEAP Q score	9.3+0.62	8.3+0.84	8.9+0.84	8.7+0.92	8.1+0.80	8.5+1.01
Mean socioeconomic status score on Modified Kuppuswamy scale	23.4+3.96	22.4+3.29	23.2+2.68	24+3.80	17.8+2.04	21.8+3.96

Caption: SLP = Speech Language Pathologist; YA = Young adults; MAA = Middle-aged adults; OA = Older adults; LEAP-Q = Language Experience and Proficiency Questionnaire.

### Procedure

**Figure1:**
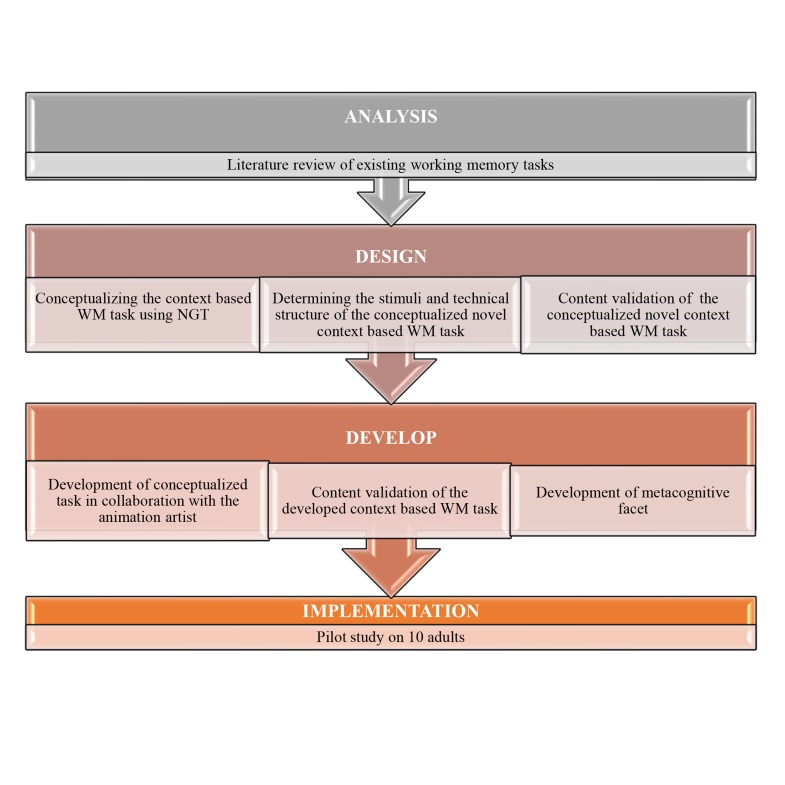
Schematic representation of the phases as per the ADDIE model

To systematically develop the novel context-based task for assessing WM with a metacognitive facet, the present study was carried out in four phases as per the ADDIE model^(28)^. Figure 1 depicts the schematic representation of each of these phases.

#### Phase 1: Analysis

The Analysis phase focused on identifying the needs and objectives for creating a context-based WM task. Literature review was carried out to examine the nature of the available WM tasks. Databases such as Pubmed, Embase and Web of Science were searched using terms like ‘WM task’, ‘Context-based WM task’, ‘Ecologically valid WM task’, ‘Everyday WM Task’, ‘Real world WM task’ to identify existing WM tasks.

#### Phase 2: Designing the context-based WM task

Designing the context-based WM task included *conceptualization of the task*, followed by *determining the stimuli and technical structure* of the conceptualized task, and *content validation* of the conceptualized context-based WM task.

The Nominal Group Technique (NGT), a structured method facilitating the generation of ideas, formulation of effective solutions, and establishment of well-founded recommendations in pursuit of best practices^(32)^ was used for conceptualizing the task. This systematic approach involves silent generation of ideas, Round Robin recording of ideas, idea clarification and consolidation, and ranking to reach an unanimously agreed-upon idea. The authors of this study served as the panel members for the NGT, with Author 1 acting as the facilitator. The NGT began with the idea generation stage where the facilitator outlined the primary objective i.e., to generate a context-based assessment task that could effectively capture the WM demands encountered in the everyday context. Each panel member was allowed 20 minutes to individually generate ideas. Following Idea Generation, the Round Robin step was employed, with each participant presenting their ideas to the panel members and facilitator simultaneously recorded the ideas presented on a white board. During the clarification step, a group discussion addressed each idea, allowing for exclusion, inclusion, modification, or grouping of similar ideas. In the final step, ranking was conducted where panel members silently ranked ideas based on cultural relevance, practical feasibility, and potential effectiveness in evaluating WM in the Indian context. Each idea was individually ranked, and the idea that received the highest averaged rank was selected to conceptualize the task.

Following the conceptualization of the task, the stimuli and technical structure of the task was determined. Author 1 and 3 designed the stimuli for the conceptualized task which included formulating the task instruction, task themes and setting, task tokens, stimuli duration and inter-stimuli interval. The technical structure of the novel context-based task was inspired from the lab-based ordering task such as reading span task^(7)^ and sentence ordering task^(6)^. The stimuli along with its technical characteristics was then reviewed by the remaining authors. Following this, the designed context-based WM task was content validated by content experts and lay experts to ensure the chosen themes, characters, and contexts were representative of everyday life and relatable to adults using a content validation form (Supplementary Material) adhering to the guidelines^(33)^.

The script was content validated for its understandability using the Patient Education Materials Assessment Tool for Printable Materials (PEMAT-P) questionnaire^(34)^ by the content experts. The Patient Education Materials Assessment Tool (PEMAT) is a reliable and valid instrument, developed by the Agency for Healthcare Research and Quality, designed to assess the understandability and actionability of patient education materials. PEMAT comprises two parts: PEMAT-P, which evaluates printable materials such as brochures and PDFs, and Patient Education Materials Assessment Tool for Audiovisual Materials (PEMAT-A/V), which assesses audiovisual materials including videos and multimedia (such as smartphone apps). Only the understandability section of the questionnaires was used to determine the understandability of the developed task.

#### Phase 3: Development of the context-based WM task

The designed context-based WM task was developed in an animated video format through collaboration with an animation artist. Adobe Illustrator and Adobe After Effects were employed for character designing, Adobe Audition for the voice-over, and Adobe Premiere Pro for the overall video production. The researchers engaged in continuous discussions with the animation artist to ensure the accurate portrayal of characters, scenes, and visual elements for each trial. Careful attention was given to ensure the voice-over used in the videos resembled an Indian accent, aiming to enhance the relatability and familiarity for the target population. Once the videos were made, it was content validated for its understandability using the PEMAT-A/V questionnaire^(34)^ by the content experts. The completed videos were integrated into the SuperLab software, a widely utilized and reliable platform for stimulus presentation and data collection in psychological experiments.

To assess metacognition, prediction and postdiction question^(35)^ were incorporated into the task. Participants were asked to provide their prediction ("Upto what level do you think you can order correctly?") and postdiction ("Upto what level do you think you have ordered correctly?") for each trial. Additionally, they were required to rate their confidence of prediction/postdiction using a 5-point Likert scale, ranging from 1 (cannot do) to 5 (can do), for two spans above and below their predicted and postdicted spans. The predicted and postdicted spans were identified as those receiving confidence ratings of 4 and 5 on the likert scale. The difference between the prediction/postdiction span and actual task performance served as the estimation discrepancy score.

#### Phase 4: Implementation of the developed context-based WM task

A pilot study was conducted on five YAs and five MAAs to assess the comprehensibility, feasibility, and practicality of the task, while also ascertaining the time required for its administration. The assessment was conducted in a well-lit, distraction-free room situated either within the departmental or residential premises. The testing environment included only the examiner and the examinee, with participants seated at a distance of 1 meter from the laptop screen. The administration of the task followed the sequence of task instruction, practice trials, prediction rating, task performance, WM span determination, and postdiction rating. Sentence Ordering (SO) and Digit Letter Ordering (DLO)^(6)^ tasks was administered to explore whether the performance spans on the novel context-based WM task correlated with established WM task measures. Qualitative feedback on task experience was gathered. Subsequently, participants also rated their anxiety levels on a scale from 0 to 10^(36)^ while doing the task. The probe questions used are displayed in table 2.

**Table 2:** Probe interview questions

Pilot study aspects	Probe questions
Comprehensibility of instructions	Did you find the activity instructions provided to the central character lengthy?
	Do you think the activity instructions are difficult to understand?
Effectiveness of Voice-over	Did you find the use of voice-over for instructions effective?
	Should voice-over be used for the entire content, or only for the activity description and completion timeline?
	In your opinion, did the voice-over enhance or hinder your understanding of the tasks?
Timing of Instructions	Do you prefer receiving detailed instructions for each trial, or having one-time instructions at the beginning of the first trial?
Necessity of clock display	Do you think the presence of a clock on each slide helpful for keeping track of time?
	Do you think displaying a clock on each slide is necessary, or could it be omitted without affecting the task completion?
Overall Task Experience	Please share your overall feedback on the task experience

### Data analysis

The results obtained from the content validation form was subjected to quantitative assessment by computing the Content Validation Index (CVI), which includes two key components: the Item-wise Content Validity Index (I-CVI) and the Scale-wise Content Validity Index (S-CVI)^(33)^. The I-CVI serve as a metric to measure the level of agreement regarding the relevance or clarity of individual items, with scores ranging from 0 to 1 is computed by determining the proportion of experts who assigned ratings of 3 or 4 to each item, divided by the total number of experts. An I-CVI exceeding 0.79 indicates that a particular item is relevant or clear, while items with an I-CVI falling between 0.70 and 0.79 indicate a need for revision, and those with an I-CVI below 0.70 suggest potential removal from the instrument. The S-CVI /Average was calculated by summing the individual I-CVI values related to relevance and dividing this sum by the total number of items within the instrument. A favorable level of content validity for the entire instrument is achieved when S-CVI /Average equaled or exceeded 0.9. Spearman’s correlation was used to assess the correlation between the performance spans on the novel developed context-based WM task and the established WM tasks (DLO and SO).

The PEMAT responses were analysed to obtain the understandability score. The understandability score calculated by adding agreed scores (excluding non-applicable items) and dividing by the total applicable items, is then multiplied by 100 to obtain the percentage value. A higher understandability score indicates greater understandability of the validated material, with a score above 70% considered acceptable^(34).^

## RESULTS

### Results of Phase 1: Analysis

Following an extensive review of literature of the existing WM tasks, it was observed that the traditional laboratory-based tasks do not capture the intricacies of everyday WM demands^(12)^. This disparity, termed the 'lab-life gap,' highlights differences between cognitive performance in lab settings and everyday life situations, raising concerns about participant engagement and the reliability of laboratory tasks^(10,11)^. Despite acknowledged disadvantages of lab-based tasks, the authors found the ordering aspect of certain tasks, such as digit span task^(5)^ and sentence ordering task^(6)^ intriguing. The existing context-based tasks often rely on virtual reality^(14)^ and are quite time consuming^(15)^, posing challenges in terms of high-end technology and ease of use. Additionally, there is a lack of context-based WM tasks specifically developed for assessing metacognition in adults within the Indian context.

Developing a context-based task that is accessible, time-efficient, and aligns with everyday WM demands, centered on the ordering aspect inspired from the laboratory-based tasks while incorporating a tailored metacognitive facet for adults in the Indian context, would be a highly beneficial initiative. Therefore, the following research question was formulated to guide the development of our task: “What are the effective ways to assess everyday WM demands encountered by adults in the Indian context while incorporating a metacognitive component?”

### Results of Phase 2: Design

The NGT was used to conceptualize the novel context-based task to assess WM. The silent generation of ideas, followed by round robin approach, led to the emergence of 10 ideas, which are provided in the table A of the Supplementary Material. Following the clarification step, six ideas were shortlisted. The results from the various steps of NGT are provided in table 3. Among the six shortlisted ideas the 'Timeline-Based Activity Ordering Task' received the highest rank and was chosen as the concept for developing a novel context-based Activity Ordering Task (AOT), with a specific focus on timelines as the organizing principle for completion.

**Table 3:** Shortlisted ideas from NGT

Sl no.	Shortlisted ideas after Step 1 and Step 2	Refined set of shortlisted ideas after Step 3 and 4	Ranks assigned
1.	Spatial-based Activity Ordering Task	Spatial-based Activity Ordering Task	2
2.	Task Importance-based Ordering	Category-based Word Recall and Ordering	6
3.	Category-based Word Recall and Ordering	Timeline-based Activity Ordering	1
4.	Recipe Ingredient Ordering Task	Daily Use Object Ordering Task	4
5.	List Creation Task	Category-based Object Ordering	5
6.	Store Section-based Object Ordering	Store Section-based Object Ordering	3
7.	Daily Use Object Ordering Task		
8.	Timeline-based Activity Ordering		
9.	Event Sequencing/Story Sequencing Tasks		
10.	Floor-based Activity Ordering		

The AOT operates as a span-based task, determining an individual's WM span progressively ranging from Level 2 involving two activities, to Level 10, incorporating ten activities. Each span includes two trials, providing a second chance if the initial attempt is unsuccessful. The task content revolves around relatable everyday themes, requiring participants to order activities for a character to be completed at different times of the day, based on instructions from family, friends, or colleagues. The primary goal is to arrange the activities in chronological order, from earliest to latest, upon receiving the prompt.

The stimuli characteristics and technical structure of the AOT were decided based on the reading span task^(7)^ and sentence ordering task^(6)^ as outlined in Table 4.

**Table 4:** Task elements

Task elements	Descriptions
Participant instruction	You'll see several scenes from everyday life in which a character is given multiple tasks to complete at different timings in a day. You must help the character order the tasks according to the timeline. The number of tasks given to the character increases as the assessment progresses. Carefully attend to each of the tasks. When the screen flashes 'Help me order,' please respond within 60 seconds. Accuracy is more important than speed; we will evaluate your response based on its correctness, not how quickly you complete it. Remember, the maximum response time is 60 seconds. Press the 'Proceed' button after completing your response.
Themes	Home/Family Errands and Work/Professional Errands
Characters	Family member, Working Parent, Homemaker, Policeman, Vegetable vendor, Railway officer, Gardener, Postman, Doctor, Peon, Bank Manager, Tailor, Milkman, Actor, Chef, Hotel Employee, Professor
Setting	Living Room, Dining Hall, Balcony, Garden, Doorway, Stable, Kitchen, School, College, Hospital, Garden, Shoot Location, Hotel, Bank, Office, Vegetable stall, Railway station, Police station, Tailoring Shop
Example of a 4-span trial token	It’s 9:00 am now and Mr. Mathew is a tailor at work. His employees and family members will approach him, each with a task for him to complete.
	Carefully attend to each of the tasks!
	You are supposed to help Mr. Mathew order the tasks in the sequence that they are to be completed based on the timeline.
	Give your response when the screen flashes “Help me order”.
	1. Sir, I have to deliver the blouse today. Please get the sewing machine fixed by 12:00 pm.
	2. Mathew, a customer will be coming to collect her dress. Please have it ready by 10:00 am.
	3. Son, there will be no electricity at home this evening. Please buy candles by 7:00 pm.
	4. Mathew, I am in your city for a week. Let’s meet today at 10:30 a.m.
Stimuli length of each activity instruction	15-20 words
Inter stimulus interval	1000 milliseconds
Progression of trials	The task goes across multiple spans starting from span 2 with two activities to be ordered and progressing to span 10 with ten activities to be ordered respectively.
Trials at each span	Each span includes two trials, offering a second chance if the first attempt is unsuccessful.
Task familiarization	The participants are initially provided with task instructions to make them familiar with the nature of the task. Subsequently, a maximum of two practice trials is administered to familiarize participants with the task's intricacies.
Response prompt slide	The concluding response prompt slide for each trial instructs participants with "help me order," indicating their goal of correctly sequencing the activities.
Response acquisition window	60 seconds
Scoring of WM span	The participant's WM span is identified as the highest successfully completed span prior to failing both tokens within a particular span. For example, if a participant accurately arranges activities up to span 4 but fails on both tokens of span 5, their recorded span is span 4.
Scoring of the metacognitive facet: Calculation of the estimation discrepancy score	The estimation discrepancy score is the difference between participant’s predicted or postdicted spans and the actual span of their task performance.
Maximum duration of the task	20-30 minutes

The content validation of the chosen themes and contexts were done using a content validation form made in accordance to the guidelines^(33)^ by 5 content experts and 15 lay experts. All the items received a CVI greater than 0.79, thus requiring no revision of the chosen themes and contexts. A S-CVI of 0.9 for lay experts and 0.93 for content experts was achieved, signifying good content validity. The details of the I-CVI and S-CVI are provided in the Supplementary Material (table B).

The content of the script was cautiously curated to ensure a balance in complexity, sentence length, and word usage. The content validation of the proofread script was done using PEMAT-P by the content experts which yielded an understandability score of 90.9%. The descriptive statistics can be found in the Supplementary Material (table C).

### Results of Phase 3: Develop

**Figure 2:**
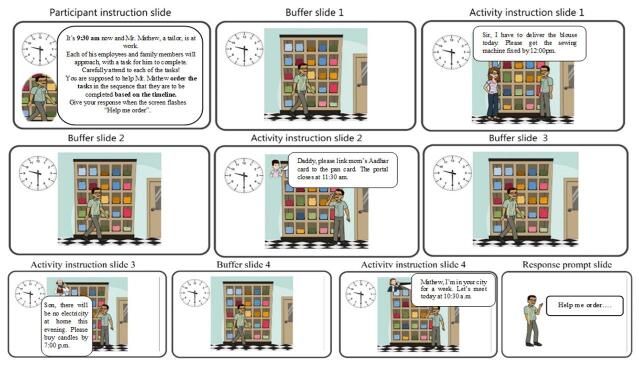
Example of Audiovisual stimuli for a 4-span trial

In the develop phase, task was constructed in an animated video format from the confirmed script in collaboration with an animation artist. The content validation of the task was done using PEMAT-AV by the content experts which yielded an understandability score of 89.6%. The descriptive statistics is outlined in the Supplementary Material (table D). An example of the stimuli developed for a trial token of a 4 span is displayed in the figure 2.

### Results of Phase 4: Implementation

**Figure 3:**
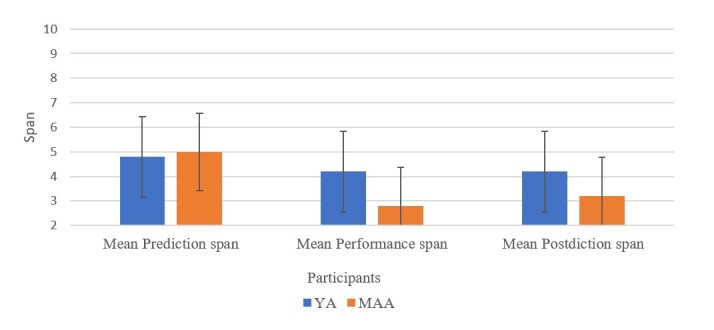
Mean prediction, performance and postdiction spans on AOT

Abbreviations: YA-Young adults; MAA -Middle-aged adults; AOT-Activity Ordering Task

Results indicate that YAs exhibited higher mean spans on performance and postdiction on the AOT compared to MAAs. MAAs demonstrated higher prediction spans when compared to YAs. The mean prediction, performance and postdiction spans of YAs and MAAs on the developed AOT are displayed in the figure 3. Correlational analysis of the performance spans on AOT with the established WM tasks indicated a significant positive correlation for DLO (r_s_ = 0.690, p = 0.027) and SO (r_s_ = 0.697, p = 0.025).

The participants took approximately 20-30 minutes to complete the entire task. The probe interview revealed unanimous agreement among participants regarding the good clarity of instructions, preferences for continuous voice-over, and mixed opinions on the necessity of a clock, ultimately highlighting a positive and engaging overall task experience. These results are provided in table 5.

**Table 5:** Results of the probe interview

Probe aspect	Results
Comprehensibility of Instructions	Participants unanimously reported that the activity instructions were exceptionally clear and straightforward, with none finding them lengthy or challenging to understand.
Effectiveness of Voice-over Instructions	All participants found the voice-over to be effective. They suggested that maintaining voice-over throughout the entire activity instruction was preferable to intermittent use, as this provided a consistent and less distracting experience, ultimately aiding their comprehension significantly.
Timing of Instructions	Regarding the timing of instructions, the majority of participants (8 out of 10) recommended displaying instructions only at the beginning of each trial. They expressed that repeated instructions within the trial could become repetitive.
Necessity of Clock Display	Regarding the inclusion of a clock on every slide, the feedback was mixed, with 6 out of 10 participants suggesting that it helped them maintain a better sense of time context.
Overall Task Experience	All participants reported an enjoyable experience during the pilot study. They noted that the tasks closely resembled activities encountered in their daily lives, allowing them to relate better. Participants reported minimal levels of subjective anxiety during task completion, with all scoring either 0 or 1 on a scale of 10.

### Discussion

The present study aimed to conceptualize and develop a novel WM assessment task with dual purposes: replicating everyday WM demands and incorporating a metacognitive facet to assess individuals' self-perceptions of their cognitive capabilities.

The ADDIE model systematically directed the development of the novel context-based everyday WM task. The Analysis phase identified the research question, the Design phase conceptualized the task and validated its content, the Develop phase transformed the script into an animated video format with additional content validation, and the Implementation phase involved the pilot testing of the developed task. The ADDIE model has found extensive application in adult education, facilitating information transfer and contributing to the creation of multimedia learning content, including animations, e-courses, modules, game-based multimedia, and interactive multimedia^(37)^. Renowned for its effectiveness in organizing health practices and patient follow-up the ADDIE model has been used across various fields^(38)^. The use of the ADDIE model in the present study aligns with its widespread applicability in diverse domains, emphasizing its adaptability in creating innovative tasks and underscoring its value in advancing research methodologies.

#### Phase 1 (Analysis): Conceptualization of the novel context-based WM task

The exploration of existing WM tasks during the analysis phase of the ADDIE framework revealed the intrinsic value of ordering tasks such as Letter Number Sequencing, SO, and Digit Ordering tasks in WM assessment, widely employed in various research contexts^(6,7)^. The ordering paradigm necessitates the reorganization of material and the maintenance of memory load during auditory information presentation, with subjects applying an overlearned ordering principle to a given memory set, retained until the ordering process completion^(5)^. While ordering tasks has good inherent benefits, a critical limitation is the absence of contextual relevance. Recognizing this, the integration of context into the well-established ordering paradigm was proposed, aligning with suggestions from the NGT panel during the Design phase. This led to the development of the novel context based AOT, a unique amalgamation of ordering task advantages and contextual relatability, surpassing conventional paradigms by capturing the crucial ordering aspect for WM assessment while enhancing participant engagement through everyday context. This novel task, requiring participants to order activities based on a provided timeline, not only engages them effectively but also mirrors the WM demands of everyday life situations, enhancing its ecological validity. Furthermore, the AOT introduces a metacognitive facet by assessing the prediction-postdiction estimation discrepancy, a well-established measure for evaluating metacognition^(24,35)^. In contrast to numerous measures relying on offline or questionnaire methods to assess metacognition, AOT employs an online method of assessing metacognition where the prediction/postdiction of one’s performance to the same task is measured in real time. Participants predict their WM span before performing the task and then postdict the highest WM span they may have achieved after performing the task. This unique feature, assessing awareness within a task context, holds the potential to offer crucial insights into everyday functioning^(22)^, presenting a dynamic and contextually rich perspective on metacognitive processes. This elevates AOT to a one-of-a-kind, multifaceted task, offering multiple benefits by providing a comprehensive assessment of everyday WM, incorporating established ordering memory principles, and delving into metacognitive processes. In essence, AOT emerges as a novel tool adept at navigating the complexities of WM assessment, effectively addressing existing ordering paradigm limitations while enriching the evaluation process.

#### Phase 2 (Design): Designing the novel conceptualized context-based WM task

Since the AOT is a context-based WM task, it was imperative to validate its content to ensure the appropriateness of the chosen themes, characters, and settings. Content validation holds paramount importance in the development of assessment tools as it verifies that the assessment items effectively capture the targeted construct being measured^(39)^. The inclusion of lay experts, ensured a user-centric validation process, enhancing the relevance and representativeness of the assessment items aligning with the goal of developing a task that resonates with the experiences of the intended participants. The themes selected, such as Home/Family Errands and Work/Professional Errands, were chosen for their reflection of common everyday scenarios, providing a broad appeal that enhances engagement, relatability, and accessibility. Diverse characters within each theme, including Family member, Working Parent, Homemaker, Gardener, Postman, Doctor, Bank Manager, and more, contribute to a comprehensive portrayal of everyday life. The inclusion of various settings like Living Room, Balcony, School/College, Hospital, and others, replicates everyday environments where people frequently run errands. These authentic settings make the tasks feel realistic and relatable, enhancing participant engagement. The high CVI values obtained justify the representativeness of the chosen themes, characters, and settings for everyday WM demands.

The designed script built upon the validated themes, characters, and settings was content validated using PEMAT-P, while the developed task was content validated using PEMAT A/V. The iterative systematic content validation carried out at different phases of the task development enhanced the precision and validity of the task^(33)^. PEMAT, a well-regarded and validated tool designed to evaluate the clarity and effectiveness of patient education materials^(34)^, is known for its internal consistency and reliability, supported by evidence of construct validity. This underscores its appropriateness for evaluating the understandability of a diverse array of printable and audiovisual materials. PEMAT's standardized and objective approach facilitated a comprehensive assessment of both the script and the developed task, in the present study. This approach aligns with established best practices in health communication research, as observed in various studies^(40)^, which emphasize the significance of creating informative materials for the targeted audience. Validation of the script and task was further reinforced, as they achieved high understandability scores of 90.9% and 89.6% respectively. Further, the involvement of an English language expert in proofreading the script was instrumental in rectifying grammatical errors, maintaining content balance for complexity and sentence structure, and ultimately improving overall comprehension.

#### Phase 3 (Develop): Developing the designed novel context-based WM task

The developed task was made in an animated video format. Computerized tasks, offering superior precision in measuring cognitive capabilities, enhanced control and specificity and diagnostic performance equivalence to traditional memory tests^(41)^, present compelling advantages over traditional paper-and-pencil methods. In aligning with these advantages, the researchers sought to enhance participant engagement and relatability by collaborating with an animation artist to create a visually appealing and meaningful task. This strategic integration of visual elements not only improved accessibility but also enriched the overall validity of the task. Successful applications of SuperLab in cognitive experiments^(42)^ provide validation of its suitability for the delivery of the AOT. The usage of SuperLab facilitated a high degree of customization in configuring task parameters to meet specific research requirements, such as the ability to skip the second token of a specific span upon participant success, enhancing the overall efficiency and adaptability of the developed novel context-based WM task.

#### Phase 4 (Implementation): Assessing the feasibility of the developed novel context-based WM task

Data trends from the implementation phase suggest potential age-related differences in performance spans on the AOT between YAs and MAAs, demonstrating the AOT's ability to detect such age-linked differences. It is crucial to emphasize that our study was primarily focused on conducting a feasibility check and hence, the age-related trends observed can be viewed as promising indicators rather than conclusive findings. Moreover, significant correlation obtained with the established WM tasks such as DLO, SO highlight the potential of AOT in tapping WM spans accurately. The observed discrepancies between performance and prediction/postdiction spans during the metacognitive facet administration demonstrate AOT's value as a tool for evaluating metacognition in addition to assessing everyday WM. Similar estimation discrepancies in prediction/postdiction paradigms have been extensively documented in the literature across a diverse range of cognitive tasks^(22,24)^. The probe interview aspects yielded positive feedback, indicating that the instructions were comprehensible, the voice-over was effective when applied consistently, timing of instructions should be primarily at the beginning of the trial and clock has to be displayed on all slides. Overall, participants found the task experience enjoyable and relatable to their daily activities. The subjective anxiety assessment scale employed in developing an ecologically valid virtual reality task for cognitive assessment in OAs^(36)^ produced comparable findings of low subjective anxiety in the present study. Participants' reports of minimal subjective anxiety while participating in the task serve as compelling evidence that the developed task is unlikely to induce anxiety and has the potential to offer an enjoyable experience for clients.

In the context of cognitive-communication, AOT could be a promising addition into the cognitive toolbox of SLPs as it bridges gaps in traditional WM assessments, offering a context-based, ecologically valid tool that incorporates a metacognitive facet. It holds the potential to evaluate the everyday communicative breakdowns contingent with WM among typically and pathologically aging adults. The systematic development process, content validation, and pilot testing reinforce the task's quality, clarity, and effectiveness for assessing WM and metacognition in Indian adults.

### Limitations and future directions

The AOT was developed based on the highest ranked idea (timeline-based activity ordering) identified through the NGT conducted during the design phase of the study. Other ideas that received relatively higher ranking (like, spatial-based activity ordering) could serve as source of inspiration for similar studies in the future, guiding the incorporation of these ideas in task development. Additionally, the present study primarily focused on Indian adults, which may limit the generalizability of findings to other populations. Cultural and demographic factors can significantly influence task performance and metacognitive aspects, highlighting the need for caution when extending the applicability of the AOT beyond this demographic. The AOT, designed to replicate the cognitive demands of everyday life, presently lacks interactive or virtual reality components. Future research could delve into integrating interactive and virtual reality elements into the AOT to enhance its ecological validity. Moreover, the feasibility check of the AOT was limited as it was conducted on a small sample size. Future studies could examine the psychometric properties of AOT to derive age specific normatives for WM span and the associated metacognitive facet, across diverse age groups and levels of education, commencing with a focus on healthy aging adults and later extending to various clinical populations. Prospective studies could also consider the time taken for ordering the activities as a variable in conjunction with the accuracy of ordering the activities.

## CONCLUSION

The AOT represents a distinctive and promising cognitive assessment tool, distinguished by several remarkable features. It offers an ecologically valid assessment of WM by replicating the cognitive demands of everyday life, enhancing its relevance in capturing participants' cognitive performance in real-world scenarios. Furthermore, the task adeptly addresses engagement challenges, ensuring active participant involvement and yielding a more accurate reflection of everyday WM. Additionally, the inclusion of a metacognitive facet allows for a deeper understanding of self-perception regarding one's WM abilities. The systematic process employed in developing the task, including iterative content validation reinforces the task's reliability and validity. Its user-friendly, computerized format seamlessly integrated into SuperLab software simplifies administration. In the forthcoming Evaluation phase, a systematic assessment of the task's psychometric properties will further underscore the utility of AOT in assessing everyday WM and related metacognition.

## Supplementary Material

Supplementary material accompanies this paper.

Table A Descriptions of shortlisted ideas from NGT

Table B Results of content validation of themes/contexts/character

Table C Descriptive statistics of the Patient Education Materials Assessment Tool for Printable Materials (PEMAT-P)

Table D Descriptive statistics of the Patient Education Materials Assessment Tool for Audiovisual Materials (PEMAT-A/V)

Table E Task composition

Content Validation Form Themes and Specific contexts/characters

This material is available as part of the online article from https://doi.org/10.1590/2317-1782/20242024041en
