# Supplementary material for: Activity ordering task: conceptualization and development of a novel context-based working memory task with a metacognitive facet
Source: Codas. 2024 Oct 11;36(6):e20240041. doi: 10.1590/2317-1782/20242024041en (PMC11529997; doi:10.1590/2317-1782/20242024041en)
Supplement: Table A [file codas-36-6-e20240041-Suppl.pdf]

SUPPLEMENTARY MATERIAL

Table A. Descriptions of shortlisted ideas from NGT

Sl no.	Idea	Description
1.	Spatial-based Activity Ordering Task	Sequence activities based on spatial proximity along the character's route or within a designated environment
2.	Task Importance-based Ordering	Prioritize tasks based on their importance
3.	Category-based Word Recall and Ordering	Recall words from a given category in an everyday conversation and arrange them in alphabetical order
4.	Recipe Ingredient Ordering Task	Order recipe ingredients based on their cooking requirements
5.	List Creation Task	Create to-do lists/shopping lists by organizing activities according to the given instructions
6.	Store Section-based Object Ordering	Order objects in a store based on their sections
7.	Daily Use Object Ordering Task	Arrange everyday objects based on their typical usage sequence in a day
8.	Timeline-based Activity Ordering	Order activities based on a provided timeline
9.	Event Sequencing/Story Sequencing Tasks	Order events or a story in the sequence of their occurrence
10.	Floor-based Activity Ordering	Order activities based on the floors on which they are to be completed

Table B. Results of content validation of themes/contexts/character

Item No.	Items	Lay experts		Content experts	
		**Experts in agreement**	**Item level CVI**	**Experts in agreement**	**Item level CVI**
	Theme 1: Home/Family Errands				
1.	The chosen theme represents everyday life experiences.	14	0.93	5	1
2.	The chosen theme is relatable to Indian adults.	13	0.87	5	1
3.	The chosen character/contexts align to the theme.	13	0.87	4	0.8
	Theme 2: Work/Professional Errands				
4.	The chosen theme represents everyday life experiences.	15	1	5	1
5.	The chosen theme is relatable to Indian adults.	14	0.93	5	1
6.	The chosen character/contexts align to the theme.	12	0.8	4	0.8
	**Scale level CVI** **0.9**			**0.93**	

Abbreviations: CVI- Content Validation Index

Table C. Descriptive statistics of the Patient Education Materials Assessment Tool for Printable Materials (PEMAT-P)

**PEMAT-P items**	**Frequency of experts (%)**		
**Understandability**<br>***Topic: Content***	**Agree**	**Disagree**	**Not Applicable**
The material makes its purpose completely evident.	100	0	0
The material does not include information or content that distracts from its purpose.	80	20	0
Topic: Word Choice & Style			
The material uses common, everyday language.	100	0	0
Medical terms are used only to familiarize the audience with the terms. When used, medical terms are defined.	0	0	100
The material uses the active voice.	100	0	0
Topic: Use of Numbers			
Numbers appearing in the material are clear and easy to understand.	100	0	0
The material does not expect the user to perform calculations.	40	40	20
Topic: Organization			
The material breaks or "chunks" information into short sections.	100	0	0
The material's sections have informative headers	20	60	20
The material presents information in a logical sequence.	100	0	0
The material provides a summary.	0	0	100
Topic: Layout & Design			
The material uses visual cues (e.g., arrows, boxes, bullets, bold, larger font, highlighting) to draw attention to key points.	100	0	0
Topic: Use of Visual Aids			
The material uses visual aids whenever they could make content more easily understood (e.g., illustration of healthy portion size).	60	0	40
The material’s visual aids reinforce rather than distract from the content.	100	0	0
The material’s visual aids have clear titles or captions.	100	0	0

The material uses illustrations and photographs that are clear and uncluttered.	100	0	0
The material uses simple tables with short and clear row and column headings.	0	0	100
**Mean Understandability Score:**		**90.9%**	

Table D. Descriptive statistics of the Patient Education Materials Assessment Tool for Audiovisual Materials (PEMAT-A/V)

**PEMAT-A/V items**	**Frequency of experts (%)**		
**Understandability** ***Topic: Content***	**Agree**	**Disagree**	**Not Applicable**
The material makes its purpose completely evident.	100	0	0
Topic: Word Choice & Style			
The material uses common, everyday language.	100	0	0
Medical terms are used only to familiarize the audience with the terms. When used, medical terms are defined.	0	0	100
The material uses the active voice.	100	0	0
Topic: Organization			
The material breaks or "chunks" information into short sections.	100	0	0
The material's sections have informative headers	20	40	40
The material presents information in a logical sequence.	100	0	0
The material provides a summary.	0	0	100
Topic: Layout & Design			
The material uses visual cues (e.g., arrows, boxes, bullets, bold, larger font, highlighting) to draw attention to key points.	80	20	0
Text on the screen is easy to read.	100	0	0
The material allows the user to hear the words clearly (e.g., not too fast, not garbled).	60	40	0
Topic: Use of Visual Aids			
The material uses illustrations and photographs that are clear and uncluttered.	100	0	0

The material uses simple tables with short and clear row and column headings.	0	0	100
**Mean Understandability Score:**		**89.6%**	

Table E. Task composition

Span	Trials	Character	Setting	No. of activities to order
Practice trial of 2-span	Practice Trial 1	Working father	Home	2
	Practice Trial 2	Policeman	Police station	2
2-span	Trial 1	Vendor	Vegetable stall	2
	Trial 2	Railway officer	Railway station	2
3-span	Trial 1	Working father	Office	3
	Trial 2	Nurse	Hospital	3
4-span	Trial 1	Gardener	Garden	4
	Trial 2	Housewife	Dining room	4
5-span	Trial 1	Postman	Post office	5
	Trial 2	Peon	Office	5
6-span	Trial 1	Tailor	Tailoring shop	6
	Trial 2	Housewife	Living room	6
7-span	Trial 1	Milkman	Stable	7
	Trial 2	Working father	House entrance	7
8-span	Trial 1	Actor	Caravan	8
	Trial 2	Working mother	Home	8
9-span	Trial 1	Working father	Living room	9
	Trial 2	Chef	Hotel Kitchen	9
10-span	Trial 1	Housewife	Hall	10
	Trial 2	Professor	College	10

Content Validation Form - Themes and Specific contexts/characters

Introduction:

We are developing a task to assess working memory using everyday life activities. Unlike traditional tasks that involve ordering digits or letters, our task focuses on everyday life scenarios to enhance relevance and engagement.

Task Overview:

In this task, participants will be presented with activities that need to be ordered for a character to be completed at different times of the day. These activities are based on instructions from family/friends or colleagues. The objective is to arrange the activities in chronological order, from earliest to latest, upon receiving the prompt "Help me order". The task comprises multiple levels, starting from Level 2 with two activities and progressing to Level 10 with ten activities. Each level includes two trials, offering a second chance if the first attempt is unsuccessful.

Themes and Contexts/characters:

To ensure the task accurately represents everyday life scenarios and captures the working memory demands encountered in daily life, we have identified two general themes and specific contexts/characters within each theme for the development of trials.

Content Validity Survey:

We appreciate your participation in this survey. The purpose of this survey is to assess the relevance of the themes and contexts/characters chosen for our task. Please rate each question on a scale of 1-5, where 1 indicates low relevance and 5 indicates high relevance. If you have any questions or need further clarification, please do not hesitate to contact us.

Theme 1: Home/Family Errands

This theme focuses on activities related to family tasks, responsibilities, social events, daily chores, and home-related activities.

a) To what extent does this theme represent everyday life experiences?
b) To what extent do you find this theme relatable to Indian adults?

c) To what extent does this theme capture the working memory demands one may encounter in everyday life?
d) Kindly propose characters and contexts that revolve around the theme of “Home/Family Errands”.

Theme 2: Work/Professional Errands

This theme focuses on activities related to office tasks, specific professions or fields, work prioritization, and professional responsibilities.

a) To what extent does this theme represent everyday life experiences?
b) To what extent do you find this theme relatable to Indian adults?
c) To what extent does this theme capture the working memory demands one may encounter in everyday life?
d) Kindly propose characters and contexts that revolve around the theme of Work/Professional Errands
